# Cognitive outcomes following unilateral magnetic resonance–guided focused ultrasound thalamotomy for essential tremor: findings from two cohorts

**DOI:** 10.1093/braincomms/fcae293

**Published:** 2024-08-30

**Authors:** Julie Petersen, Josh McGough, Georgia Gopinath, Nadia Scantlebury, Richa Tripathi, Cheryl Brandmeir, Silina Z Boshmaf, Nicholas J Brandmeir, Isabella J Sewell, Peter E Konrad, Agessandro Abrahao, Ann Murray, Benjamin Lam, Manish Ranjan, Clement Hamani, Jessica Frey, Camryn Rohringer, Melissa McSweeney, James J Mahoney, Michael L Schwartz, Ali Rezai, Nir Lipsman, David M Scarisbrick, Jennifer S Rabin

**Affiliations:** Rockefeller Neuroscience Institute, West Virginia University School of Medicine, Morgantown, WV 26505, USA; Department of Psychiatry, Harvard Medical School at Beth Israel Deaconess Medical Center, Boston, MA 02215, USA; Rockefeller Neuroscience Institute, West Virginia University School of Medicine, Morgantown, WV 26505, USA; Hurvitz Brain Sciences Program, Sunnybrook Research Institute, Toronto, ON, Canada M4N 3M5; Harquail Centre for Neuromodulation, Sunnybrook Research Institute, Toronto, ON, Canada M4N 3M5; Jean and Paul Amos PD and Movement Disorders Program, Department of Neurology, Emory University School of Medicine, Atlanta, GA 30307, USA; Rockefeller Neuroscience Institute, West Virginia University School of Medicine, Morgantown, WV 26505, USA; Hurvitz Brain Sciences Program, Sunnybrook Research Institute, Toronto, ON, Canada M4N 3M5; Department of Neuroscience, West Virginia University School of Medicine, Rockefeller Neuroscience Institute, Morgantown, WV 26505, USA; Department of Neurosurgery, West Virginia University School of Medicine, Rockefeller Neuroscience Institute, Morgantown, WV 26505, USA; Hurvitz Brain Sciences Program, Sunnybrook Research Institute, Toronto, ON, Canada M4N 3M5; Department of Neuroscience, West Virginia University School of Medicine, Rockefeller Neuroscience Institute, Morgantown, WV 26505, USA; Department of Neurosurgery, West Virginia University School of Medicine, Rockefeller Neuroscience Institute, Morgantown, WV 26505, USA; Hurvitz Brain Sciences Program, Sunnybrook Research Institute, Toronto, ON, Canada M4N 3M5; Harquail Centre for Neuromodulation, Sunnybrook Research Institute, Toronto, ON, Canada M4N 3M5; Division of Neurology, Department of Medicine, Sunnybrook Health Sciences Centre, University of Toronto, Toronto, ON, Canada M4N 3M5; Department of Neurology, West Virginia University School of Medicine, Rockefeller Neuroscience Institute, Morgantown, WV 26505, USA; Hurvitz Brain Sciences Program, Sunnybrook Research Institute, Toronto, ON, Canada M4N 3M5; Division of Neurology, Department of Medicine, Sunnybrook Health Sciences Centre, University of Toronto, Toronto, ON, Canada M4N 3M5; Department of Neurosurgery, West Virginia University School of Medicine, Rockefeller Neuroscience Institute, Morgantown, WV 26505, USA; Hurvitz Brain Sciences Program, Sunnybrook Research Institute, Toronto, ON, Canada M4N 3M5; Harquail Centre for Neuromodulation, Sunnybrook Research Institute, Toronto, ON, Canada M4N 3M5; Division of Neurosurgery, Department of Surgery, Sunnybrook Health Sciences Centre, University of Toronto, Toronto, ON, Canada M4N 3M5; Department of Neurology, West Virginia University School of Medicine, Rockefeller Neuroscience Institute, Morgantown, WV 26505, USA; Hurvitz Brain Sciences Program, Sunnybrook Research Institute, Toronto, ON, Canada M4N 3M5; Hurvitz Brain Sciences Program, Sunnybrook Research Institute, Toronto, ON, Canada M4N 3M5; Rockefeller Neuroscience Institute, West Virginia University School of Medicine, Morgantown, WV 26505, USA; Department of Neuroscience, West Virginia University School of Medicine, Rockefeller Neuroscience Institute, Morgantown, WV 26505, USA; Department of Behavioral Medicine and Psychiatry, West Virginia University School of Medicine, Rockefeller Neuroscience Institute, Morgantown, WV 26505, USA; Hurvitz Brain Sciences Program, Sunnybrook Research Institute, Toronto, ON, Canada M4N 3M5; Division of Neurosurgery, Department of Surgery, Sunnybrook Health Sciences Centre, University of Toronto, Toronto, ON, Canada M4N 3M5; Department of Neuroscience, West Virginia University School of Medicine, Rockefeller Neuroscience Institute, Morgantown, WV 26505, USA; Department of Neurosurgery, West Virginia University School of Medicine, Rockefeller Neuroscience Institute, Morgantown, WV 26505, USA; Hurvitz Brain Sciences Program, Sunnybrook Research Institute, Toronto, ON, Canada M4N 3M5; Harquail Centre for Neuromodulation, Sunnybrook Research Institute, Toronto, ON, Canada M4N 3M5; Division of Neurosurgery, Department of Surgery, Sunnybrook Health Sciences Centre, University of Toronto, Toronto, ON, Canada M4N 3M5; Department of Psychiatry, Sunnybrook Health Sciences Centre, University of Toronto, Toronto, ON, Canada M4N 3M5; Department of Neuroscience, West Virginia University School of Medicine, Rockefeller Neuroscience Institute, Morgantown, WV 26505, USA; Department of Behavioral Medicine and Psychiatry, West Virginia University School of Medicine, Rockefeller Neuroscience Institute, Morgantown, WV 26505, USA; Hurvitz Brain Sciences Program, Sunnybrook Research Institute, Toronto, ON, Canada M4N 3M5; Harquail Centre for Neuromodulation, Sunnybrook Research Institute, Toronto, ON, Canada M4N 3M5; Division of Neurology, Department of Medicine, Sunnybrook Health Sciences Centre, University of Toronto, Toronto, ON, Canada M4N 3M5; Rehabilitation Sciences Institute, University of Toronto, Toronto, ON, Canada M5G 1V7

**Keywords:** magnetic resonance–guided focused ultrasound, cognition, thalamotomy, essential tremor, neuropsychology

## Abstract

Magnetic resonance–guided, focused ultrasound thalamotomy is a neurosurgical treatment for refractory essential tremor. This study examined cognitive outcomes following unilateral magnetic resonance–guided, focused ultrasound thalamotomy, targeting the ventral intermediate nucleus of the thalamus for essential tremor. The research was conducted at two sites: Sunnybrook Research Institute in Toronto, Canada, and West Virginia University School of Medicine Rockefeller Neuroscience Institute in West Virginia, USA. The study focused on cognitive changes at both the group and individual levels. Patients with refractory essential tremor completed cognitive testing before and after magnetic resonance–guided, focused ultrasound thalamotomy at both sites. The cognitive testing assessed domains of attention, processing speed, working memory, executive function, language and learning/memory. Postoperative changes in cognition were examined using paired *t*-tests and Wilcoxon signed-rank tests, as appropriate. Reliable change indices were calculated to assess clinically significant changes at the individual level. A total of 33 patients from Toronto and 22 patients from West Virginia were included. Following magnetic resonance–guided, focused ultrasound thalamotomy, there was a significant reduction in tremor severity in both cohorts. At the group level, there were no significant declines in postoperative cognitive performance in either cohort. The reliable change analyses revealed some variability at the individual level, with most patients maintaining stable performance or showing improvement. Taken together, the results from these two independent cohorts demonstrate that unilateral magnetic resonance–guided, focused ultrasound thalamotomy significantly reduces tremor severity without negatively impacting cognition at both the group and individual levels, highlighting the cognitive safety of magnetic resonance–guided focused ultrasound thalamotomy for essential tremor.

## Teaser

Petersen *et al*. examined cognitive outcomes following unilateral focused ultrasound thalamotomy for essential tremor in two academic medical centres. In both cohorts, tremor severity improved without negatively impacting cognition at both the group and individual levels. These findings highlight the cognitive safety of this procedure.

## Graphical Abstract

**Graphical Abstract:**
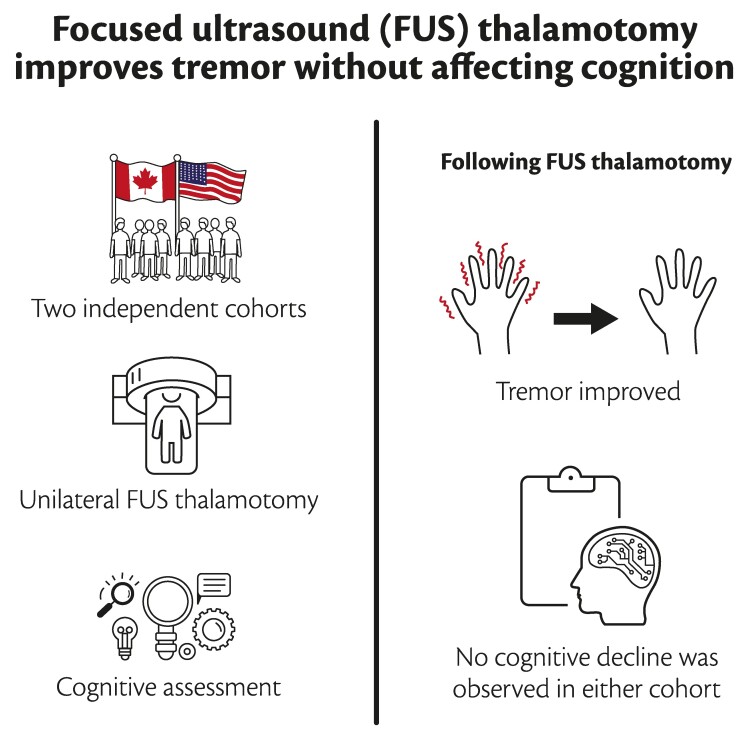

## Introduction

Medication-refractory essential tremor (ET) can severely impact daily tasks requiring fine motor dexterity, such as writing, eating, drinking and dressing.^1^ When pharmacotherapy treatments fail, surgical interventions targeting the ventral intermediate nucleus of the thalamus (VIM) may be considered. Magnetic resonance–guided, focused ultrasound (MRgFUS) is a relatively new, minimally invasive neurosurgical procedure that effectively reduces tremor by lesioning the VIM.^2^

Existing knowledge about cognitive outcomes following unilateral MRgFUS VIM thalamotomy for ET is limited. To our knowledge, only four published studies have investigated cognitive changes following unilateral MRgFUS thalamotomy in patients with ET, with sample sizes ranging from 9 to 22.^3-6^ These studies collectively indicate no significant cognitive decline at the group level across a range of cognitive domains, including attention, processing speed, executive function, language and learning/memory. While group-level analyses provide important insights into the cognitive safety of a procedure, they may mask individual variations.

To address this gap, the present study used reliable change indices (RCIs)^7^ to assess meaningful individual-level cognitive changes following unilateral MRgFUS thalamotomy targeting the VIM in patients with medication-refractory ET. RCIs provide thresholds for determining meaningful changes for test scores, distinguishing ‘true’ changes from those attributable to measurement error.^7^ We conducted our evaluations across two independent cohorts located in Toronto, Canada, and West Virginia, USA, with the primary objective of generating robust data about the cognitive safety of MRgFUS thalamotomy for ET.

## Materials and methods

### Participants

Data were collected from Sunnybrook Research Institute in Toronto, Canada, and West Virginia University School of Medicine Rockefeller Neuroscience Institute (WVU-RNI) in West Virginia, USA. The Institutional Review Boards at both sites approved this study (Toronto: REB# 2106, WVU-RNI: IRB# 2211672546). All patients provided informed consent prior to study procedures.

Participants in the present study were individuals with medication-refractory ET who underwent unilateral MRgFUS VIM thalamotomy and had cognitive data available at baseline and follow-up. Diagnosis of ET was made by a movement disorder neurologist based on criteria outlined by the International Parkinson and Movement Disorder Society.^8^ Cognitive assessments were performed at the Toronto site from August 2020 to July 2023, and at the WVU-RNI site from February 2020 to August 2022.

To be eligible for MRgFUS thalamotomy, individuals with ET were required to have significant disability caused by tremor. Medication-refractory status was determined by at least two failed trials of pharmacotherapies for tremor management. Patients were not treated if they had contraindications to MRI. In the Toronto cohort, most patients had a skull density ratio (SDR) above 0.44, within the range of 0.24–0.7. In the WVU-RNI cohort, patients had an SDR above 0.40.

### Tremor assessment

In both cohorts, tremor severity was assessed using the Clinical Rating Scale for Tremor (CRST).^9^ In the Toronto cohort, the CRST score reflected the sum of Parts A and B for the treated hand, excluding the handwriting item. In the WVU-RNI cohort, the CRST score reflected the total score across all subscales.

### Cognitive assessment

Participants in both cohorts underwent cognitive evaluations before and after MRgFUS thalamotomy. In the Toronto cohort, cognitive testing was performed as part of a research study, while in the WVU-RNI cohort, cognitive testing was part of clinical care. Follow-up testing occurred ∼4 months after treatment in the Toronto cohort and ∼6.5 months after treatment in the WVU-RNI cohort.

The cognitive test battery in both cohorts included standardized neuropsychological tests evaluating domains of attention, processing speed, working memory, executive function, language and learning/memory (see Supplementary Table 1 for a list of tests). Cognitive test scores were standardized based on age using test-specific normative data. Patients who were outside the age range of available normative data were excluded from the respective analyses (10 test scores were omitted across both cohorts). Select measures were additionally corrected for education and race.

### MRgFUS thalamotomy

In both cohorts, unilateral MRgFUS thalamotomy was performed during a single session using the ExAblate Neuro 4000 system. Imaging and thermal mapping were performed using a 3-T MRI. The VIM was targeted during initial sonication based on stereotactic planning and a preoperative MRI. The MRgFUS VIM thalamotomy procedure has been described in detail previously by Lipsman *et al*.^10^

### Clinical diagnosis

The patients in the Toronto cohort did not undergo cognitive diagnostic evaluations to identify neurocognitive disorders. In the WVU-RNI cohort, the presence of a neurocognitive disorder was determined through a clinical consensus conference involving an interdisciplinary team. The team included experts from neuropsychology, neurology, neurosurgery, neuroradiology and physical therapy. Diagnoses of mild and major neurocognitive disorders were made using the Diagnostic and Statistical Manual of Mental Disorders, Fifth Edition (DSM-5) criteria.^11^

### Statistical analyses

Group-level changes: Before performing group-level analyses, standardized cognitive test scores were transformed into *z*-scores. This transformation ensured consistency across reported results and facilitated meaningful comparisons across different cognitive domains. For all tests, higher *z*-scores reflected better performance. Group-level changes were assessed with paired *t*-tests and Wilcoxon signed-rank tests, as appropriate.^12^ The Benjamini–Hochberg procedure with a false discovery rate of 0.05 was applied to account for multiple comparisons.^13^ Effect sizes for paired *t*-tests were computed using Cohen’s *d*, while effect sizes for Wilcoxon signed-rank tests were calculated as the *r* estimate.^14^ Statistical analyses were performed using IBM SPSS Statistics version 29.0.2.0. Raincloud plots visualizing raw tremor and cognitive scores were generated using the *PtitPrince* package in Python.^15^

Individual-level changes: RCIs were computed to assess changes in cognition at the individual level. RCIs provide thresholds for determining meaningful changes for test scores, distinguishing ‘true’ changes from those attributable to measurement error.^7^ RCIs were computed using the Jacobson–Truax method^16^ implemented with the *JTRCI* package in R.^17^ The scores were classified as a reliable decline when the RCI was ≤−1.96, a reliable improvement when the RCI was ≥1.96, and considered to have no reliable change when the RCI fell between −1.96 and 1.96.

For cognitive test scores, RCIs were computed using parameters derived from the normative sample, which included the test reliability coefficient (*r_xx_*) and standard deviation (SD). A second analysis used the *r_xx_* of the normative sample, but the SD of the present sample. We employed this latter method due to concerns about potential differences between parameters in our clinical sample and those in the normative sample. However, a comparison of the two methods revealed similar patterns of reliable change. For brevity, we only report the results using the initial RCI method, with all parameters derived from the normative sample. In the WVU-RNI cohort, scores from the list learning memory test (California Verbal Learning Test, Second edition, Short form) were excluded from RCI analyses due to the use of different test forms at baseline and follow-up, as well as the lack of published test-retest reliability information for this test. Although different versions of the Hopkins Verbal Learning Test–Revised were used in the Toronto cohort, data from these versions were retained for RCI analyses due to the equivalent reliability estimates for the two forms used (Forms 1 and 4).^18^ Violin plots illustrating the distribution of RCIs were created using the Seaborn package in Python.^19^

## Results

### Participant characteristics

Table 1 presents the baseline characteristics of patients with ET from each cohort. In the Toronto cohort, there were 33 patients (76% male). Among them, 32 patients underwent ablation to the dominant hemisphere (5 left-handed patients). One patient opted for ablation to the non-dominant hemisphere due to severe tremor affecting left-hand function. The median time interval between the baseline assessment and treatment with MRgFUS was 11 days [interquartile range (IQR) = 7 days]. The median time interval between treatment with MRgFUS and the follow-up assessment was 108 days (IQR = 41 days).

**Table 1:** Demographic and clinical baseline characteristics

Characteristic		Toronto cohort (*n* = 33)	WVU-RNI cohort (*n* = 22)
Age (years)		69 (10)	73 (8)
Education (years)		15 (3)	12 (4)^a^
Sex, *n* (%)	Male	25 (76)	11 (50)
	Female	8 (24)	11 (50)
Race/ethnicity, *n* (%)	Asian	1 (3)	0 (0)
	Non-Hispanic Black/African American	3 (9)	0 (0)
	Non-Hispanic White	29 (88)	22 (100)
Tremor duration (years)		24 (24)^a^	22 (41)^a^
Handedness, *n* (%)	Right	28 (85)	16 (73)
	Left	5 (15)	6 (27)
Side of ablation, *n* (%)	Right	6 (18)	6 (27)
	Left	27 (82)	16 (73)
Baseline cognitive status, *n* (%)	Normal		8 (36)
	Mild neurocognitive impairment		11 (50)
	Major neurocognitive impairment		3 (14)
Baseline/MRgFUS interval (days)		11 (7)^a^	94 (85)^a^
MRgFUS/follow-up interval (days)		108 (41)^a^	201 (51)^a^

Continuous data are reported as mean (SD) if normally distributed and as median (IQR) if the distribution is non-normal. MRgFUS, magnetic resonance–guided focused ultrasound; WVU-RNI, West Virginia University School of Medicine Rockefeller Neuroscience Institute. ^a^A non-normal distribution.

In the WVU-RNI cohort, there were 22 patients (50% male). All patients in this cohort underwent ablation to the dominant hemisphere (six left-handed patients). The median time interval between the baseline cognitive assessment and treatment with MRgFUS was 94 days (IQR = 85 days). The median time interval between the MRgFUS procedure and the follow-up cognitive assessment was 201 days (IQR = 51).

### Group-level analyses

**Figure 1:**
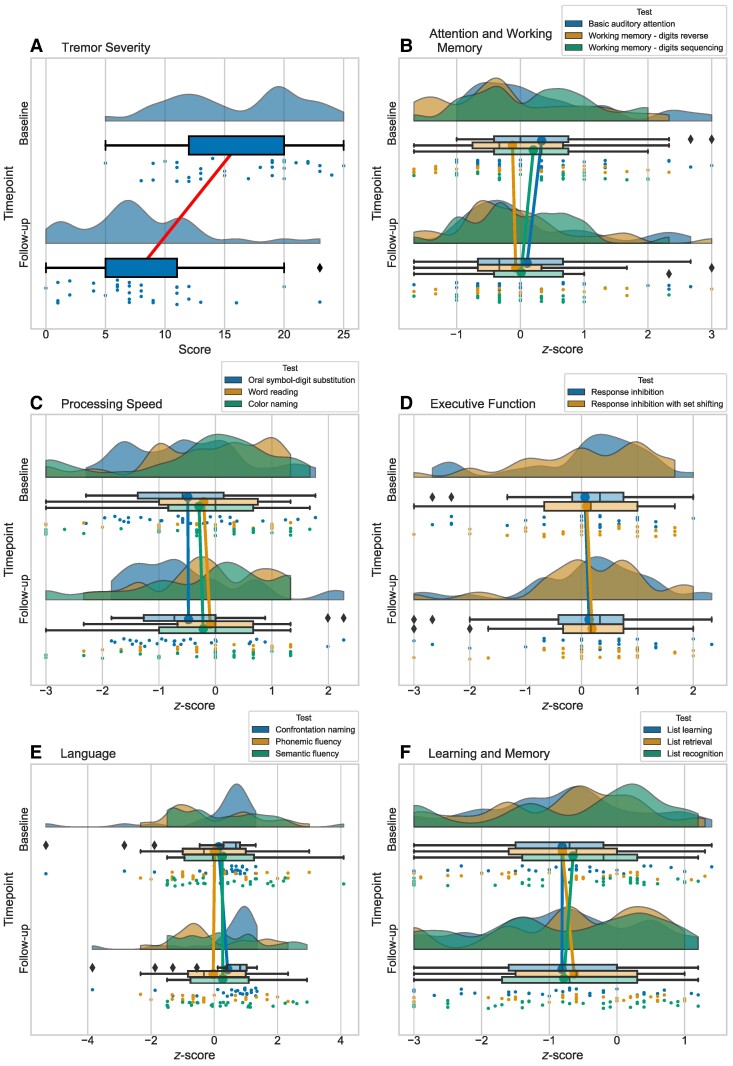
**Tremor and cognitive scores at baseline and follow-up in the Toronto cohort.** Raincloud plots showing the distribution of tremor and cognitive scores. Cognitive test scores were converted to *z*-scores. Each plot includes: unmirrored violin plots (probability density functions); boxplots displaying medians and IQRs, with whiskers at 1.5 × IQR and outliers denoted by diamonds; lines connecting the mean values at baseline and follow-up; and individual data points, which are vertically jittered. The plots represent: (**A**) tremor severity, (**B**) attention and working memory, (**C**) processing speed, (**D**) executive function, (**E**) language and (**F**) learning and memory. Details on these measures are available in Supplementary Table 1.

In the Toronto cohort, we observed a significant reduction in tremor severity from baseline to follow-up (*Z* = −4.69, *P* < 0.001, *r* = −0.82). In terms of cognition, there were no significant declines observed across any of the cognitive test scores. There was a modest but significant improvement on a measure of confrontation naming (*Z* = −3.16, *p*_cor_ = 0.03, *r* = −0.63). Figure 1 illustrates the baseline and postoperative follow-up scores in the Toronto cohort, with the pre/post comparisons summarized in Supplementary Table 2.

**Figure 2:**
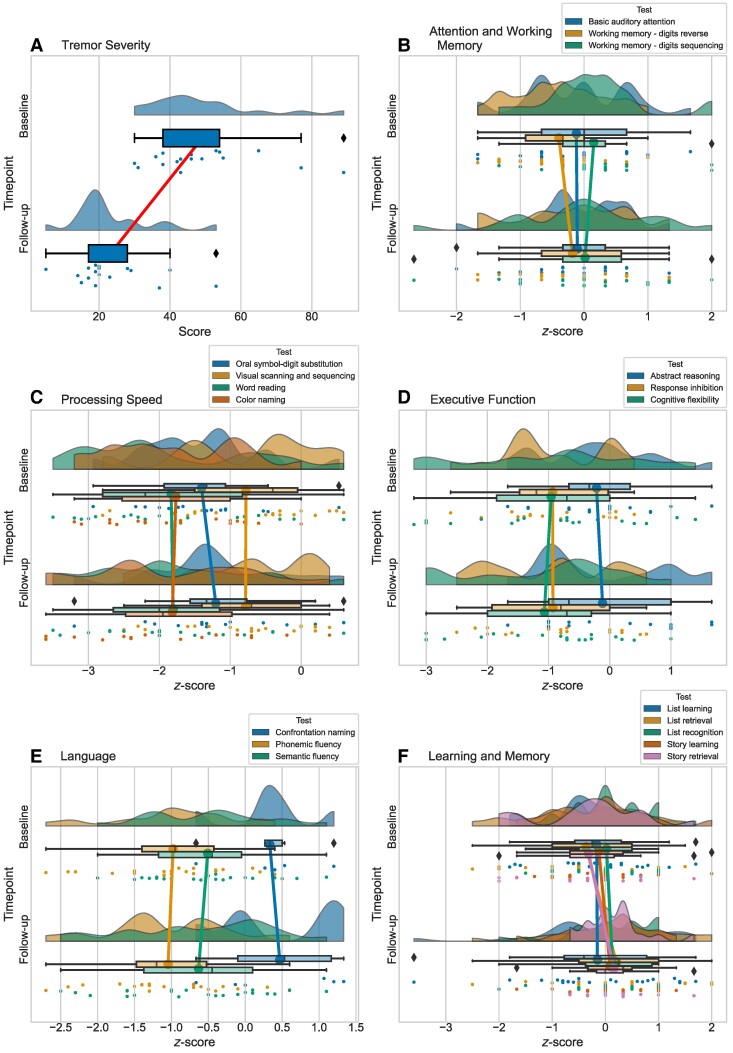
**Tremor and cognitive scores at baseline and follow-up in the WVU-RNI cohort**. Raincloud plots showing the distribution of tremor and cognitive scores. Cognitive test scores were converted to *z*-scores. Each plot includes: unmirrored violin plots (probability density functions); boxplots displaying medians and IQRs, with whiskers at 1.5 × IQR and outliers denoted by diamonds; lines connecting the mean values at baseline and follow-up; and individual data points, which are vertically jittered. The plots represent: (**A**) tremor severity, (**B**) attention and working memory, (**C**) processing speed, (**D**) executive function, (**E**) language and (**F**) learning and memory. Details on these measures are available in Supplementary Table 1. WVU-RNI, West Virginia University School of Medicine Rockefeller Neuroscience Institute.

Like the Toronto cohort, there was a significant reduction in tremor severity in the WVU-RNI cohort following MRgFUS (*Z* = −3.62, *P* < 0.001, *r* = −0.62). With respect to cognition, none of the cognitive test scores significantly changed from baseline to follow-up. Figure 2 illustrates the baseline and postoperative follow-up scores in the WVU-RNI cohort, with the pre/post comparisons summarized in Supplementary Table 3.

### Individual-level analyses

**Figure 3:**
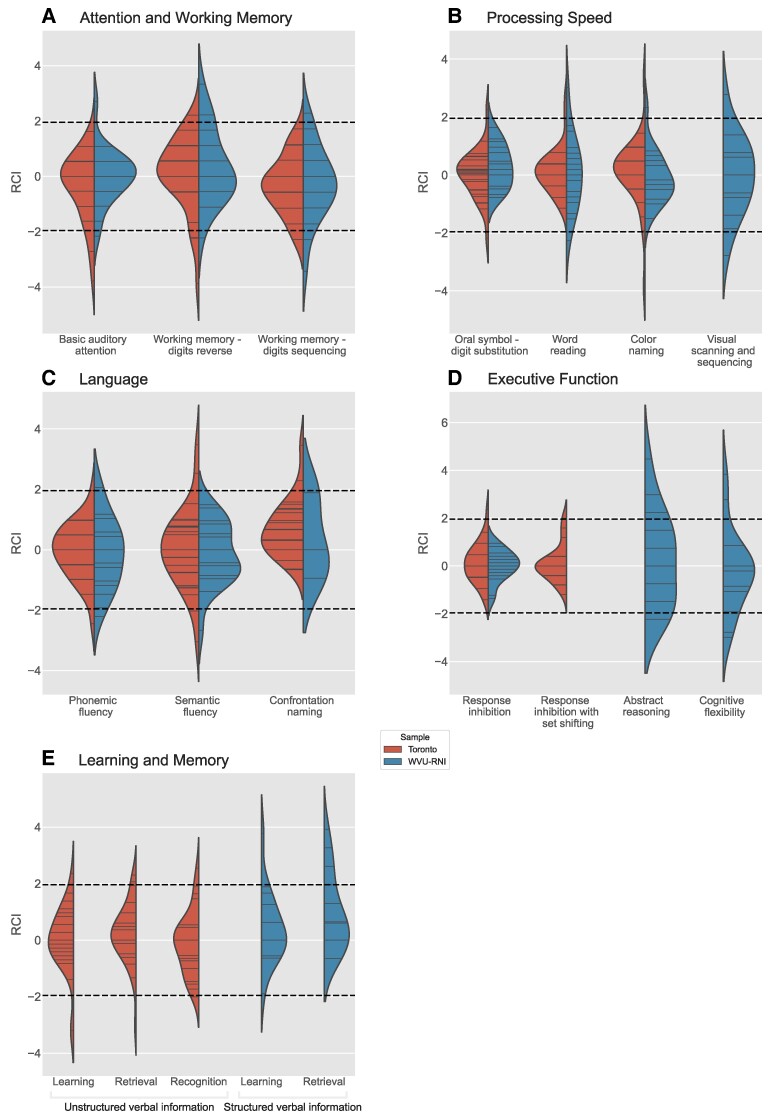
**Violin plots showing kernel density estimates of the reliable change distributions of cognitive test scores by site.** Individual patient data points are represented as sticks within the distribution. RCIs were calculated using standardized scores. Dashed lines are plotted at RCI values of −1.96 and 1.96, with values below −1.96 indicating a reliable decline and values above 1.96 indicating a reliable improvement. **(A)** Attention and working memory measures, **(B)** processing speed, **(C)** executive function, **(D)** language and **(E)** learning and memory. Details on these measures are available in Supplementary Table 1. WVU-RNI, West Virginia University School of Medicine Rockefeller Neuroscience Institute.

Across all patients in both cohorts (*n* = 55), 24 patients showed reliable improvement on at least one cognitive measure and 24 patients showed reliable decline on at least one cognitive measure (Table 2, Fig. 3). Notably, no patient declined on more than two tests. Six patients demonstrated both improvement and decline on at least one measure. Furthermore, 13 patients did not show reliable change on any measure. A close inspection of the data revealed no consistent pattern of improvement or decline across cognitive domains.

**Table 2:** Reliable change in cognitive performances

		Toronto cohort			WVU-RNI cohort		
Domain	Ability	Declined	No change	Improved	Declined	No change	Improved
Attention and working memory	Basic auditory attention	3 (9)	29 (91)	0 (0)	1 (5)	20 (90)	1 (5)
	Working memory—digits reverse	3 (9)	28 (88)	1 (3)	1 (5)	17 (77)	4 (18)
	Working memory—digits sequencing	2 (7)	26 (93)	0 (0)	2 (9)	19 (86)	1 (5)
Processing speed	Oral symbol-digit substitution	1 (4)	26 (92)	1 (4)	0 (0)	19 (100)	0 (0)
	Visual scanning and sequencing				1 (5)	17 (90)	1 (5)
	Word reading	0 (0)	31 (97)	1 (3)	1 (6)	16 (88)	1 (6)
	Colour naming	1 (3)	29 (94)	1 (3)	0 (0)	17 (94)	1 (6)
Executive function	Response inhibition	0 (0)	31 (97)	1 (3)	0 (0)	18 (100)	0 (0)
	Response inhibition with set shifting	0 (0)	30 (94)	2 (6)			
	Abstract reasoning				3 (18)	11 (64)	3 (18)
	Cognitive flexibility				2 (11)	15 (78)	2 (11)
Language	Confrontation naming	0 (0)	23 (92)	2 (8)	0 (0)	6 (100)	0 (0)
	Phonemic fluency	1 (3)	29 (94)	1 (3)	1 (5)	19 (86)	2 (9)
	Semantic fluency	2 (6.5)	26 (87)	2 (6.5)	1 (5)	21 (95)	0 (0)
Learning and memory	List learning	2 (6)	30 (91)	1 (3)			
	List retrieval	1 (3)	30 (91)	2 (6)			
	List recognition	1 (3)	31 (94)	1 (3)			
	Story learning				0 (0)	18 (95)	1 (5)
	Story retrieval				0 (0)	14 (74)	5 (26)

Reliable change is reported as *n* (% of cohort). Reliable change indices for tests of list-learning and story memory were calculated separately with form-specific test reliability metrics. Combined totals of both structured verbal learning and memory forms are reported above. Additional information about each measure can be found in Supplementary Table 1. WVU-RNI, West Virginia University School of Medicine Rockefeller Neuroscience Institute.

As previously noted, cognitive diagnoses were not assigned to patients in the Toronto cohort. In the WVU-RNI cohort, at baseline, 8 (36%) patients had normal cognition, 11 (50%) patients met the criteria for mild neurocognitive disorder, and 3 (14%) patients met the criteria for major neurocognitive disorder. Across these diagnostic groups, the pattern of reliable change was generally consistent, although some variability was observed. Overall, patients in each diagnostic group showed improvement on some cognitive measures, experienced decline on no more than two measures, and maintained stable performance on at least six measures. The maximum number of tests showing reliable decline in any one patient was two, which was observed in two patients with normal cognition and one patient with a major neurocognitive disorder.

## Discussion

The present longitudinal study examined the cognitive effects of unilateral MRgFUS VIM thalamotomy in patients with medication-refractory ET, using data from two independent cohorts: one in Toronto, Canada, and the other in West Virginia, USA. A unique aspect of this study was the use of both group- and individual-level analyses to assess postoperative cognitive changes. Across both cohorts, unilateral MRgFUS VIM thalamotomy significantly reduced tremor severity without negatively affecting cognition. While some variability was observed at the individual level, most patients displayed stable or improved postoperative cognitive performance. These findings contribute to the growing body of literature suggesting that unilateral MRgFUS VIM thalamotomy is a cognitively safe treatment option for patients with medication-refractory ET.

Our group-level findings are consistent with several previous studies suggesting that unilateral MRgFUS VIM thalamotomy is not associated with cognitive decline.^3-6,20^ In our study, no significant group-level postoperative changes were observed across any of the cognitive measures. The only exception was confrontation naming, which showed an improvement in the Toronto cohort, but not in the WVU-RNI cohort. However, this finding should be interpreted with caution, as reliable improvement was observed in only two patients in the Toronto cohort, while performance in all other patients remained stable.

There was some intraindividual variability in our individual-level findings. While most cognitive test scores remained stable or improved postoperatively, some showed reliable declines. Importantly, these changes were not confined to any single cognitive domain. These declines may be due to several factors, including normal neuropsychological variability,^21,22^ the underlying disease process associated with ET,^23,24^ or surgical factors, such as larger lesion volumes or lesion locations. We were unable to confirm the latter possibility, as this information was not consistently tracked across all study patients.

In the WVU-RNI cohort, we examined the impact of baseline cognitive impairment on postoperative outcomes. Among the patients, 11 (50%) had mild neurocognitive disorder and three (14%) had major neurocognitive disorder. Consistent with previous findings,^6^ patients with preexisting cognitive disorders did not exhibit a higher risk of postoperative decline compared with those with normal cognition. However, caution is warranted in interpreting these results, as our analysis was limited to a single cohort with a relatively small number of cognitively impaired individuals.

The group-level results from this study are consistent with existing literature on deep brain stimulation (DBS) for ET, which generally shows that VIM stimulation is safe from a cognitive standpoint.^25,26^ While our individual-level analyses did not show consistent declines in any cognitive domain, VIM DBS studies have reported mild but consistent postoperative declines in executive function, episodic memory and verbal fluency at the individual level.^25,27,28^ This suggests that VIM MRgFUS may be a cognitively safer alternative, although further research is needed to confirm this.

The strengths of the present study lie in the replication of findings across two independent cohorts. Importantly, consistency in results persisted despite differences in geographical setting (urban versus rural), type of study (research-based versus part of clinical care), education levels of participants, cognitive tests employed and timing of the follow-up visits. However, there were several limitations. First, both cohorts were relatively small and based on convenience samples. The small sample sizes limited statistical power, making it challenging to detect small or moderate effects. Second, the data set consisted of patients who returned for follow-up testing, which could lead to selection bias. Third, the sample composition of patients in both cohorts was predominantly White, which may limit the generalizability of our findings. Future studies should replicate these findings in more diverse cohorts.

## Conclusion

Unilateral MRgFUS VIM thalamotomy is a cognitively safe and effective treatment for reducing tremor in individuals with medication-refractory ET. Our findings indicate no postoperative changes at the group level. While there was some variability at the individual level, there was no consistent decline observed. Therefore, patients with ET and their healthcare providers can have confidence in the cognitive safety of unilateral MRgFUS VIM thalamotomy when considering it as a treatment option.

## Supplementary Material

fcae293_Supplementary_Data

## Data Availability

The data are available upon request.
