# Supplementary material for: Cognitive outcomes following unilateral magnetic resonance–guided focused ultrasound thalamotomy for essential tremor: findings from two cohorts
Source: Brain Commun. 2024 Aug 30;6(5):fcae293. doi: 10.1093/braincomms/fcae293 (PMC11406546; doi:10.1093/braincomms/fcae293)
Supplement: fcae293_Supplementary_Data [file fcae293_supplementary_data.pdf]

Supplementary Material

Table of Contents

Supplementary Table 1. Measures across cohorts 2

Supplemental Table 2. Cognition and tremor severity across time in the Toronto cohort 3

Supplemental Table 3: Cognition and tremor severity across time within the West Virginia University School of Medicine – Rockefeller Neuroscience Institute cohort 4

Scripts 5

References 6

Supplementary Table 1. Measures across cohorts

Domain	Ability	Toronto Cohort Measures	WVU-RNI Cohort Measures
Tremor severity	--	CRST (1) – Parts A and B of the hand contralateral to the treated hemisphere	CRST – Total Score
Attention and working memory	Basic auditory attention	WAIS-IV (2) – Digit Span Forward	WAIS-IV – Digit Span Forward
	Working memory – digits reverse	WAIS-IV – Digit Span Backward	WAIS-IV – Digit Span Backward
	Working memory – digits sequencing	WAIS-IV – Digit Span Sequencing	WAIS-IV – Digit Span Sequencing
Processing speed	Oral symbol-digit substitution	SDMT (3) – Oral	SDMT – Oral
	Visual scanning and sequencing	--	Trail Making Test (4) – Part A
	Word reading	D-KEFS (5) – CWIT – Word Reading	SCWT (6) – Word
	Color naming	D-KEFS – CWIT – Color Naming	SCWT – Color
Executive function	Response inhibition	D-KEFS – CWIT – Inhibition	SCWT – Color-Word
	Response inhibition with set shifting	D-KEFS – CWIT – Inhibition/ Switching	--
	Abstract reasoning	--	WAIS-IV – Matrix Reasoning
	Cognitive flexibility	--	Trail Making Test – Part B
Language	Confrontation naming	Boston Naming Test (7)	--
	Phonemic fluency	FAS (baseline) and BHR (follow-up)	FAS
	Semantic fluency	Animals	Animals
Learning and memory	List learning	HVLT-R (8) – Total Recall	CVLT-II (9) – Trials 1-5 Free Recall
	List retrieval	HVLT-R – Delayed Recall	CVLT-II – Long Delay Free Recall
	List recognition	HVLT-R – Recognition Discrimination Index	CVLT-II – Discriminability
	Story learning	--	WMS-IV (10) – Logical Memory I
	Story retrieval	--	WMS-IV – Logical Memory II

- *Abbreviations*: CRST, Clinical Rating Scale for Tremor; CVLT-II, California Verbal Learning Test, Second Edition; CWIT, Color-Word Interference Test; D-KEFS, Delis-Kaplan Executive Function System; HVLT-R, Hopkins Verbal Learning Test-Revised; SCWT, Stroop Color-Word Test; SDMT, Symbol Digit Modalities Test; WAIS-IV, Wechsler Adult Intelligence Scale, Fourth Edition; WMS-IV, Wechsler Memory Scale, Fourth Edition.
- All cognitive test scores were age-corrected and standardized using test-specific normative samples. All language measures and scores from TMT were additionally corrected for race and education using available normative reference groups.(11)
- Toronto patients were administered HVLT-R Form 1 at baseline and Form 4 at follow-up.
- WVU-RNI patients were administered either the CVLT-II standard form ($N = 5$ at baseline), alternate form ($N = 5$ at follow-up), or short form (N=17 at both baseline and follow-up).
- WVU-RNI patients received either the WMS-IV adult ($N = 4$) or older adult ($N = 18$) version, depending on age.
- SDMT scores from 4 Toronto patients and 6 WVU-RNI patients were excluded from analyses due to their age exceeding that of available normative data.

Supplemental Table 2. Cognition and tremor severity across time in the Toronto cohort

Domain	Ability		Baseline		Follow-up		Test Statistic	p_{corr}	Effect Size
		N	*M*	*SD*	*M*	*SD*			
Tremor severity	--	33	16.12	5.31	7.91	5.22	$Z = -4.69$	<0.001*	$r = -0.82$
Attention and working memory	Basic auditory attention	32	0.33	1.09	0.10	1.07	$Z = -1.54$	0.46	$r = -0.27$
	Working memory – digits reverse	32	-0.13	1.10	-0.07	1.00	$Z = -0.66$	0.76	$r = -0.12$
	Working memory – digits sequencing	28	0.20	0.95	0.01	0.84	$t(27) = 1.67$	0.46	$d = 0.32$
Processing speed	Oral symbol-digit substitution	28	-0.49	1.06	-0.48	1.06	$Z = -0.31$	0.87	$r = -0.06$
	Word reading	32	-0.21	1.19	-0.09	0.99	$Z = -0.61$	0.76	$r = -0.11$
	Color naming	31	-0.29	1.34	-0.22	1.24	$Z = -1.17$	0.60	$r = -0.21$
Executive function	Response inhibition	32	0.63	1.18	0.14	1.17	$Z = -0.59$	0.75	$r = -0.10$
	Response inhibition and set shifting	32	0.09	1.16	0.19	1.09	$t(31) = -0.83$	0.61	$d = -0.15$
Language	Confrontation naming	25	0.14	1.47	0.42	1.19	$Z = -3.16$	0.02*	$r = -0.63$
	Phonemic fluency	31	0.01	1.39	-0.03	1.28	$Z = -0.32$	0.76	$r = -0.06$
	Semantic fluency	30	0.25	1.41	0.27	1.31	$Z = -0.14$	0.89	$r = -0.01$
Learning and memory	List learning	33	-0.81	1.12	-0.81	1.15	$t(32) = 0.02$	0.89	$d = -0.01$
	List retrieval	33	-0.81	1.14	-0.63	1.19	$Z = -1.35$	0.53	$r = -0.24$
	List recognition	33	-0.65	1.33	-0.78	1.27	$Z = -1.07$	0.87	$r = -0.19$

*Significant difference detected between baseline and follow-up in confrontation naming ($p_{corr} = 0.02$) and tremor severity ($p < 0.001$), such that performance on confrontation naming and tremor severity improved.

Supplemental Table 3: Cognition and tremor severity across time within the West Virginia University School of Medicine – Rockefeller Neuroscience Institute cohort

Domain	Ability	N	Baseline M	Baseline SD	Follow-up M	Follow-up SD	Test Statistic	p_{corr}	Effect Size
Tremor severity	--	19	48.65	14.69	22.21	11.23	$Z = -3.62$	<0.001*	$r = -0.62$
Attention and working memory	Basic auditory attention	22	-0.12	0.85	-0.11	0.76	$t(21) = -0.12$	0.96	$d = -0.03$
	Working memory – digits reverse	22	-0.39	0.72	-0.18	0.91	$t(21) = -1.23$	0.69	$d = -0.26$
	Working memory – digits sequencing	22	0.15	0.79	0.02	1.04	$Z = -0.75$	0.87	$r = -0.11$
Processing speed	Oral symbol-digit substitution	15	-1.40	0.86	-1.20	0.96	$t(15) = -1.49$	0.60	$d = -0.39$
	Visual scanning and sequencing	19	-0.78	1.02	-0.78	0.91	$t(19) = 0.03$	0.60	$d = 0.01$
	Word reading	18	-1.83	1.21	-1.81	1.10	$t(17) = -0.17$	0.98	$d = -0.04$
	Color naming	18	-1.77	0.93	-1.82	1.16	$t(17) = 0.40$	0.87	$d = 0.09$
Executive function	Abstract reasoning	17	-0.22	0.91	-0.12	1.08	$Z = -0.23$	0.96	$r = -0.04$
	Response inhibition	18	-0.93	0.85	-0.93	1.02	$t(17) = -0.05$	0.96	$d = -0.01$
	Cognitive flexibility	19	-0.96	1.39	-1.07	1.24	$t(18) = 0.62$	0.94	$d = 0.14$
Language	Confrontation naming	6	0.33	0.60	0.47	0.84	$t(5) = -0.60$	0.58	$d = 0.24$
	Phonemic fluency	22	-0.98	0.82	-1.05	0.83	$t(21) = 0.40$	0.94	$d = 0.09$
	Semantic fluency	22	-0.51	0.79	-0.63	0.98	$t(21) = 0.60$	0.96	$d = 0.13$
Learning and memory	List learning	22	-0.16	0.88	-0.15	1.23	$t(21) = -0.06$	0.96	$d = -0.01$
	List retrieval	22	-0.36	1.06	0.21	1.13	$t(21) = -2.91$	0.08	$d = -0.62$
	List recognition	22	0.02	0.70	0.14	0.82	$Z = -0.93$	0.81	$r = -0.14$
	Story learning	19	-0.12	0.88	0.09	0.74	$t(18) = -1.39$	0.60	$d = -0.32$
	Story retrieval	19	-0.32	0.98	0.16	0.56	$t(18) = -2.96$	0.08	$d = -0.68$

*Significant difference detected between baseline and follow-up tremor severity ($p < 0.001$), such that tremor severity improved postoperatively

Scripts

Scripts for plotting and RCI analyses can be found here: https://shorturl.at/qQ2HZ

References

1. Fahn S, Tolosa E, Marín C. Clinical rating scale for tremor. Parkinson's disease and movement disorders. 1993;2:271-80.
2. Wechsler D. Wechsler adult intelligence scale–Fourth Edition (WAIS–IV). San Antonio, TX: NCS Pearson. 2008;22(498):1.
3. Smith A. Symbol digit modalities test: Western psychological services Los Angeles; 1973.
4. Reitan R. Trail making test: Manual for administration and scoring: Reitan Neuropsychology Laboratory. Back to cited text. 1992(48).
5. Delis DC, Kaplan E, Kramer JH. Delis-Kaplan executive function system. 2001.
6. Golden CJ, Freshwater SM. Stroop color and word test. 1978.
7. Kaplan E, Goodglass H, Weintraub S. Boston naming test. 2001.
8. Shapiro AM, Benedict RH, Schretlen D, Brandt J. Construct and concurrent validity of the Hopkins Verbal Learning Test–revised. The Clinical Neuropsychologist. 1999;13(3):348-58.
9. Delis DC, Kramer JH, Kaplan E, Ober BA. California Verbal Learning Test. Assessment. 1987.
10. Drozdick LW, Raiford SE, Wahlstrom D, Weiss LG. The Wechsler Adult Intelligence Scale—Fourth Edition and the Wechsler Memory Scale—Fourth Edition. 2018.
11. Heaton RK. Revised comprehensive norms for an expanded Halstead-Reitan Battery: Demographically adjusted neuropsychological norms for African American and Caucasian adults, professional manual: Psychological Assessment Resources; 2004.
